# Editorial Chit-Chat

**Published:** 1878-10

**Authors:** 


### Editorial Chit-Chat.

HOSPITALS AND HARD TIMES.—Our foreign medical journals say that the London hospitals are partially closed for want of means to keep them open.

---

ITHACA.—Dr. Schliemann has obtained permission from the Greek government to excavate in Ithaca. Strange the *Journal* keeps so quiet about it. Hope the Dr. will not undermine the University.

---

PERPLEXED CORONERS.—At a recent nitro-glycerine explosion in Bradford, Pa., four men were blown to atoms. The latest information from the scene of the catastrophe announces that the coroners were waiting for the men to come down, that an inquest might be held.

---

THAT TENT.—We gave but half material enough for the roof of the tent figured in the last BISTOURY. It will require twice the quantity mentioned. The other measurements are correct. If you use a fly, six strips, 17½ feet long, will be needed.

---

ATTRACTIVE, not only that, but adhesive likewise, is the Opera House. Seated in one of the tilting chairs for an hour, one needs to be pried loose, or must leave a portion of his coat fastened to the back of the chair. A little shellac and alcohol would remedy the trouble.

---

A DANGEROUS NUISANCE.—At the foot of State street, in this city, empties a sewer, under one end of the Beach building, that has become a very serious affair. All the filth of the sewer settles immediately under the building, from which noxious gases arise, having caused at least two very serious cases of illness among occupants of the premises. There has been too much delay in abating the nuisance. The Board of Health should remedy it immediately, and decide afterward, whose duty it is to pay the bill.

---

NEW STEAMER.—Many of our readers will be surprised to learn that a new and elegant screw steamer has been launched upon the waters of the Chemung river, and at most any hour of the day, may be seen plying about amongst the less pretentious craft of the river. She is not a steamer of the first class nor has her tonnage been registered, so far as we can learn, but she is entitled to the name steamer as there is no diminutive termination to apply. She was built, equipped and manned by several young men of this city, and to all seeming (we have not been a passenger) reflects praise upon the ingenuity of the builders.

The dimensions are about those of an ordinary steam yawl, and she has made about eight miles an hour.

---

WELL ANSWERED.—The Williamsport *Gazette & Bulletin*, recently published the particulars of an accident, in which the following occurred:

At Walnut street and Park avenue, Miss Turley was thrown out. She struck a fence with her *back* which injured the spinal column, and made it necessary to carry her *back* to her home.

A correspondent then asked: "What was done with the balance of the lady?"

To which the editor promptly replied:

"Such a question! Why, the 'balance of the lady' was lost as she fell out of the buggy."

---

LUSUS NATURÆ.—Who ever heard a mill dam? Who ever saw a rope walk? Similar conundrums have recently been propounded by one paragrapher to another, but the one we are about to ask is not of a frivolous character, though the form of question implies it.

Who ever saw a white red squirrel? The answer is, "We have."

A friend returning from the country a few weeks ago, brought with him and presented to us a young squirrel, one of a family of red squirrels, which was of a creamy white hue. The little Albino differed in no other respect physically from his tawny brethren, but was not equally beloved by his parents. Mother squirrel conceived a great aversion to her strange young one, and chased him from the nest at an early age with garrulous threats. A trap was set for him and he bit. Since when he degenerated into a common mountebank and daily performed in his wheel to juvenile crowds.

Several days ago he up and died in an epileptic spasm, superinduced, no doubt, from violent exercise. He will now change his wire cage for a glass case.

---

BEAUTIFUL SCULPTURE.—Upon the rise of ground in the southeast corner of Woodlawn Cemetery, at a turn of the Avenue, lies the family lot of General A. S. Diven. Upon it, he has caused to be erected a monument that attracts lovers of the beautiful often to the place.

It consists of a granite base, upon which rests a marble pedestal, about seven feet high, containing the epitaphs. Above this, a cap, adorned with sculptured wreaths of flowers, and upon which is beautifully poised a statue, so faultlessly modeled as to excite our wonder and admiration.

The figure is of that disciple whom our Lord so loved, St. John. The inscription upon the pedestal below affording a clue to the sentiment which the sculptor so successfully embodied :

"I heard
a voice from heaven
saying unto me
WRITE,
from henceforth
blessed are the
dead who die in
THE LORD."

The figure is, perhaps, seven feet tall, faultlessly proportioned, the left foot, upon which the weight of the body rests, slightly advanced, the face inclined upward, eyes directed heavenward, the left hand holding a book, in easy attitude, the right grasping a pencil with the hand and arm raised as if waiting the conclusion of the command; the figure beautifully illustrating the attitude of expectancy. But it is the countenance which the artist has transfigured: the expression of hope predominates, but about it is the calm majesty of holiness, worthy to be worn even by a Saint.

In this beautiful work of art Gen. Diven has at once satisfied one's æsthetic and sympathetic nature. A more graceful tribute to the dead could not well be imagined.

---

THE RACES.—On the 10th day of September, the Elmira Driving Park opened its gates for a series of races to extend over four days. The State Fair was in full blast during that week, and thus a double attraction was offered to strangers to pay us a visit. This meeting was also looked forward to by nearly every member of the community, who noted the entries, as a season offering exceptional opportunities for witnessing some really good races. The time has come when the practical benefit derived from careful breeding and training of horses, is universally acknowledged. Then, too, most of the early day coarse, and often immoral, adjuncts of a race track are banished, and in consequence the spectators of to-day are of a superior grade. There was every reason to believe that the proceedings would be conducted in a fair and honorable manner. Under the auspices of this favorable public sentiment the Fall meeting opened. Now that it is concluded, instead of expressing pleasure and bestowing approbation upon what followed, we are obliged to stigmatize the proceedings as an unmitigated bore. It is not worth while to enter into the many disappointments to which real lovers of legitimate racing were subjected to. It is enough to say, that of all disgraceful features, the most vexatious one was the interminable scoring which was allowed. Even the daily press lost patience and loudly rated such stupidity or such double-dealing. No matter which it was, it was equally intolerable to the spectators.

The Pool Stand was the objective point of interest, and any horse would be shamed, ( could he understand ) to feel that his gifts were prostituted to cater to the depraved appetite for gambling, which alone animated the throng of sharpers and black-legs, which crowded around it. Bah! the very air reeked with the fumes of whisky and bad cigars.

"Reform it, gentlemen, reform it altogether," else will you soon see an honorable and gentlemanly sport, pass into the hands of those who have made it a tool readily fashioned for the gambler's hand.

---

THE STATE FAIR.—The annual exhibition of the New York State Agricultural Society was held in this city from the 9th to the 13th day, inclusive, of September last. For many years following the institution of these fairs, they proved to be not only sources of pecuniary profit to the society, but they were also esteemed and supported by all who desired to bring into public notice any improvement, invention or contrivance which had for its object the saving of labor or the perfection of its results. Formerly the attendance was composed mostly of those who desired to learn and adopt any thing or suggestion, which, on examination of the products of thought or experience of others, might seem valuable. Latterly, however, it is not to be denied that those engaged in commercial pursuits, attend seldom, if at all. The grounds are given, in the main, to venders or manufacturers, principally speculators in agricultural implements. There is generally a crowd, be it great or less, according to the weather, drawn together by idle curiosity.

This last exhibition was a singularly good one and was so pronounced by every careful observer, yet the attendance was comparatively light. The reasons for the lack of interest are various. Some say that the Philadelphia Exposition of 1876 was of such gigantic proportions, that it created a distaste in the minds of the people for any show upon a lesser scale. The contrast was certainly present to the mind of every spectator. Some say the spirit of Communism and its absurd hatred of labor-saving machines is at the root of it. Some see in it an indication of a decline in love of agricultural pursuits. There surely is a weakening some where.

Rain fell during all of the last day, and it virtually put a quietus on further proceedings. The same bedraggled crowd of sight-seers; a motley concomitant of every Fair, all day long thronged the highways and railroads in its mournful exodus from our city, many reproaching themselves that they allowed a flaming placard and a silly desire to "see the crowd" to tempt them from a comfortable home.

If there is not a moral to be drawn, there is at least a conclusion to be arrived at, viz:

State Fairs have outgrown their usefulness, and the people of New York have outgrown State Fairs.

---

THE POWER OF MUSIC.—The world is dark to a blind man. This is clear to our readers, but they do not understand, nor can they believe how extravagant is the expression of joy and gratitude that gushes up from the heart when first vision is restored to a blind man and he again beholds the light of day. One day, not very long ago, the hero of our tale, who is a venerable old man of the patriarchal school, was in sore need of surgical aid, a cataract had formed on one eye and the sight of the other was fast waning. He applied at the proper place for relief and was told that the growth upon his eye could be removed. Some idea of the operation he evidently had and was fully convinced that it was one involving most excruciating pain. He was led to the operating chair, therefore, suffering from great agitation. Our friend was a man of huge proportions, but his bulk failed to supply the place of nerve, for his fear reduced him to a most pitiable and childish condition. His thin quavering voice, in indistinct utterance, seemed to plead with the surgeon for mercy; his nervous fingers closed upon the arms of the chair with a grip like a vise. The Doctor began his professional duties in a quiet way, entertaining the old man with a few remarks about the weather; meantime the nervous patient hung on as if resolved not to go alone, if go he must, but take the chair with him. At the moment when he had braced himself to the very pitch of fortitude, the Doctor informed him that the cataract was off. Then his hair stood erect, his grip relaxed, and with a sigh that would do credit to a blast furnace, he roared, "Gracious goodness, is it possible? Why, I never felt it a bit." For a time he was placed in a dark room; then, one night he was sufficiently recovered to go into the men's ward. His joy knew no bounds. His sight was returning, and our scanty English vocabulary was too limited to supply him with expressions of gratitude to his benefactor.

Next morning, at an early hour, the words of the popular song, "The Little Old Log Cabin in the Lane," backed by a powerful pair of lungs, rolled in volumed cadence through the silent halls. The very air was rife with sounds which permeated every nook and chamber of the building. The startled sleepers sat upright in frightened expectation, while waves of stentorian melody rolled and broke against the trembling chamber doors.—The resident physician, in scant attire, fought his way through the sonorous current in the passage, gasping for breath. He opened the door.

"Hold on! Hold on there! Some of the people in the neighborhood want to sleep an hour or two yet."

The voice of the gray-haired warbler waned, broke and retreated to its cave and the door closed behind the intruder. A thought struck him.

Oh Doc! Oh Doc! he whispered at the top of his voice.

"Well," said the Doctor, rushing back, "What's the matter now?"

"Oh! nothin'," said the ancient singer of ballads, "Only, thinks I, how durned lucky it is that I dunno only one varse."

So saying, he turned placidly around in his chair to force on a pair of No. 14 socks, "so 's to git ready for brekfust."

---

THE DOBSON.—Probably none but residents of this city will know at first sight what the subject of this paragraph is. To the best of our ability we will explain: The dobson is of the order of reptiles. He has a black, scaly back, in plates like a lobster's tail, with a small pair of ice tongs for a head. He grows to the average length of four inches, and has three pairs of legs. His home is in some running stream where he hides under the stones until the time arrives when he is transformed and becomes another insect. At first, he looks, that is when dressed in his dobson suit, like a big, India rubber "thousand-legged" worm. His bite is not necessarily fatal.

So much for his appearance. Naturally, the query follows: "What is such an ugly beast good for, and why do they call him a dobson?"

We can briefly dispose of the latter question, though by no means to your satisfaction. Being of an inquiring turn of mind, we have upon several occasions catechised dealers in the dobson as to why they should give him such a simple name, when his complex structure should entitle him to something more difficult to pronounce. A Latin or Greek word compounded with several prepositions, would be more applicable to the squirming fellow. But, it seems scientific men can not do him justice. Severest cross-examination only elicits the information, "That's what they all call 'em."

Even his origin has not been traced. He is in fact, as little known as that other gay bird, the Dodo, or any reptile of the Preadamite period. He is long, thin, dark, fierce, inscrutable, and his mysterious life and metamorphosis really entitle him to the enjoyment of that vague and shadowy future which is allotted him by the ordinary dwellers on the river side. "He goes," say they, "inter a sort uv a bug that flies 'round on wings. And yet he don't fly much nuther."

We are glad to chronicle the change with so much accuracy.

But to return to the main point. "What is a dobson?" We are sorry that your sedentary habit of life has not led you to his better acquaintance. We address all you who in a search after the great unknown, have not yet found that which alone can satisfy your craving; you who have hitherto been content to lure the finny tribe with clams, worms, the squids, grasshoppers and the like. And you, oh fly-fishermen, pause with uplifted arms, remove your many colored flies and impale upon your hook a squirming dobson.

Let writers for *Forest and Stream* vent all their jealous spleen upon our many legged friend, he can run away from them all; let manufacturers of artificial bait, take down their signs; let angleworms squirm with envy; let the great green grasshoppers hop on, we'll have none of them. The dobson is king.

To all who would inveigle the gamy bass, of which our river has now a fair supply, or deceive the gay pickerel, or the fancy sun-fish, let me describe the method of the dobson's capture. "Put money in thy purse," then saunter out into the street. Hail the first bare-legged youngster you meet.

"Sonny, can you get me some dobson's?"
"Yes, sir,"

"Bring fifty to my house in half an hour."
"Yes, sir."

The lad knows where a supply is kept on hand by some enterprising dobson merchant, and by the time you have securely corked your lunch basket, you will find him on the horse-block, with your purchase. The price fluctuates, but the present quotation is $1.00 per hundred. Now you are equipped. Don't be afraid to take the main streets to the river; if you should meet any of your friends don't be ashamed or afraid of their strictures on your vagabond propensities, it is their off day. They probably have a note to meet, and can't get away to go. One more word: Now that you have stopped, take 'em, when you bait 'em by by the nape of the neck or they will make their nippers meet under your skin. A well authenticated story comes to us of a fellow up the river who was fishing. Some careless fish went to fooling around a dobson on the hook, came too near and was seized and held until the fisherman landed him. Believe it or not, you have dobson's choice.

There is one more important view to take of the case. If there is a "down easter" among us, let him take out a patent and found a stock company, "The Chemung Society for the Propagation of Dobsons," for instance. A new branch of commercial industry will thus open up in our midst. It will give new impetus to the State Hatcheries for fish, and it will solve the labor problem, for by division of labor, trade is advanced. Finally, it will throw hundreds of reckless dobson speculators out of employment, and in like proportion will increase the daily attendance of our declining primary schools.

---

FLOWER HUNTING.—The Spring and Fall furnish us with the most delightful of wild flowers, in search of which lovers of them never tire. Early in the spring, we have the *trailing arbutus*, of delicate hue and grateful perfume, excursions after which afford the rambler pleasure and pleasant exhileration.

Now is the season in which that rare plant with its exquisitely fringed flowers—the *fringed gentian*—can be found, if you are fortunate enough to know its hiding places. So seldom is this delightful flower met with and so beautiful is it when found, that it will repay one in exploring for it. Seek for it among the undergrowth on our hilltops, and when you find it, please let us know that we may join you in the gathering So far, we have heard of but two ladies who have succeeded in finding this coy little flower, and who were kind enough to conduct us to its hiding place. Over hills and through the fields, we rode,—a merry party on horseback, jumping ditches, leaping over fences, scrambling through the scrubby underbrush until our eyes were delighted with a view of the pretty blue blossoms that hied us on, and which have been ornamenting our sanctum table for the past two weeks.

